# Transcriptional effects of influenza virus M1 and NP gene expression in DF-1 cells and their immunogenicity in cognate B21 chickens

**DOI:** 10.1186/s12917-026-05433-8

**Published:** 2026-04-07

**Authors:** Sun-Min Ahn, Ho-Won Kim, Minji Kim, So-Jeong Lim, Hyeon-Jin Sa, Seung-Ji Kim, Hana Park, Kang-Seuk Choi, Hyuk-Joon Kwon

**Affiliations:** 1 Laboratory of Poultry Medicine, Department of Farm Animal Medicine, College of Veterinary Medicine, BK21 PLUS for Veterinary Science, Seoul National University, Seoul, 88026 Republic of Korea; 2 Research Institute for Veterinary Science, College of Veterinary Medicine, Seoul National University, Seoul, 08826 Republic of Korea; 3 Laboratory of Avian Diseases, College of Veterinary Medicine, Seoul National University, Seoul, 08826 Republic of Korea; 4 Department of Biomedical Sciences, College of Medicine, Seoul National University, Seoul, 03080 Republic of Korea; 5 GeNiner Inc., Seoul, 08826 Republic of Korea

**Keywords:** Influenza A virus, Matrix protein 1 (M1), Nucleoprotein (NP), Transcriptomics, MHC-matched model, Host gene expression, Cell-based vaccine

## Abstract

**Background:**

Although the influenza A virus (IAV) matrix protein 1 (M1) and nucleoprotein (NP) are attractive targets for cross-protective vaccines, their independent effects on host cell transcriptomes and subsequent immunogenicity in histocompatible hosts remain largely unexplored. Here, we utilized a previously established histocompatible DF-1 cell and a cognate B21 chicken (DF-1/B21) platform to characterize these interactions. Stable DF-1 cell lines expressing M1 or NP were analyzed via transcriptomic profiling and subsequently used as single-antigen vaccines.

**Results:**

Transcriptional analysis revealed distinct host signatures: M1 expression was primarily associated with alterations in membrane-associated and cytoskeletal pathways. In contrast, NP expression was linked to the broad downregulation of DNA replication and cell cycle regulators, actin network reorganization, and the modulation of early innate signaling without inducing a full interferon response. Functional assays corroborated these findings, showing that despite higher viral susceptibility in M1-expressing cells, NP expression established a potent antiviral state characterized by reduced viral replication and an increase in pro-inflammatory (TNF-α, IL-6) and immune-recruiting (IL-15, IL-12p40) cytokines in vitro. NP, but not M1, was also spontaneously released into culture supernatants. In vivo, both vaccines elicited measurable humoral responses and reduced viral shedding upon H9N2 challenge. Furthermore, distinct shifts in peripheral CD8⁺/CD4⁺ ratios post-challenge indicated that both antigens effectively modulated cellular immunity.

**Conclusions:**

Collectively, these findings validate the DF-1/B21 system as a versatile platform for dissecting host–pathogen interactions. By effectively bridging cellular transcriptomics with systemic immunity, this histocompatible model offers a valuable framework for the broad evaluation of avian immunogens and the rational design of future poultry vaccines.

**Supplementary Information:**

The online version contains supplementary material available at 10.1186/s12917-026-05433-8.

## Background

 Influenza A virus (IAV) remains a persistent threat to both public health and the poultry industry due to its zoonotic potential and the challenges in developing universal vaccines [1–3]. In veterinary medicine, control strategies have primarily relied on whole inactivated virus (WIV) vaccines to mitigate disease burden [4]. While effective, WIV vaccines present antigens in a fixed stoichiometry, which masks the specific immunogenic contributions of individual viral proteins [5, 6]. Consequently, the independent effects of conserved internal proteins on host cellular machinery and their subsequent capacity to elicit protective immunity remain difficult to evaluate within the context of conventional vaccination.

Among the internal viral proteins, nucleoprotein (NP) and matrix protein 1 (M1) are attractive targets for cross-protective immunity. NP is highly conserved and serves as a primary target for cytotoxic T lymphocytes (CTLs) via MHC class I–restricted presentation [7–10]. In contrast, M1 is crucial for virion assembly and structural integrity, though its role in modulating host immune responses is less clearly defined [11, 12]. Importantly, while the structural functions of these proteins are well documented, their intrinsic impact on the host cell transcriptome—particularly how they reprogram cellular pathways to favor viral replication or prime immune signaling—remains incompletely characterized. Understanding these cellular signatures is critical, as they likely dictate the quality of the subsequent adaptive immune response.

Current gene-based approaches (e.g., DNA or mRNA vaccines) allow for the study of single antigens; however, most mechanistic studies have been conducted in mammalian systems [13–15]. These findings are not directly distinct to avian hosts due to fundamental species differences in antigen processing, lymphoid architecture, and MHC repertoires [16–18]. The lack of histocompatible avian models has hindered our ability to predict how specific viral genes modulate immunity in poultry, which are central to influenza ecology. Therefore, a homologous system is required to accurately link cellular antigen expression with systemic immune outcomes.

To address this gap, we employed a genetically matched host–cell platform comprising the B21 MHC haplotype chicken line and DF-1 cells, a continuous chicken fibroblast line sharing a similar genetic background. Building on our previous work demonstrating the immunogenicity of HA-expressing DF-1 cells in B21 chickens [19], we extended this strategy to the internal proteins of the H9N2 influenza virus. In this study, we established stable DF-1 cell lines expressing either M1 or NP to elucidate antigen-specific transcriptional reprogramming in vitro and to evaluate their immunogenicity in vivo.

By leveraging this histocompatible cell and animal (DF-1/B21) platform, we aimed to compare how the distinct cellular environments shaped by M1 and NP expression influence humoral and cellular immune responses. This study provides a comprehensive analysis of host–virus interactions, demonstrating that the DF-1/B21 platform is a valuable framework for defining the immunological correlates of viral internal proteins and for guiding the rational design of next-generation avian vaccines.

## Results

### Distinct host transcriptional responses induced by M1 and NP gene expression in DF-1 cells

**Fig. 1:**
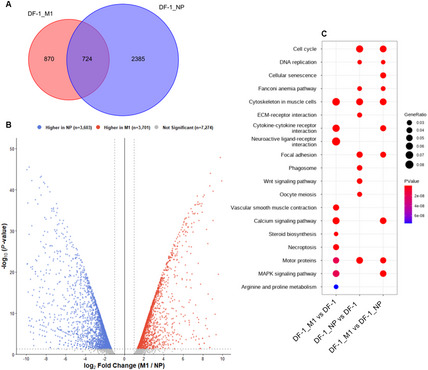
Comparative transcriptomic analysis of DF-1 cells expressing Influenza virus M1 and NP. **A** Venn diagram illustrating the number of unique and shared differentially expressed genes (DEGs) in M1- and NP-expressing cells compared to the negative control, DF-1 (FDR < 0.05, |log2FC| ≥ 1). **B** Volcano plot showing the direct transcriptomic comparison between M1 and NP (total *n* = 14,578). Positive log2FC values (red) indicate genes up-regulated in M1 (*n* = 3,701), while negative values (blue) indicate genes up-regulated in NP (*n* = 3,603) relative to M1 (nominal *p* < 0.05, |log2FC| ≥ 1). The symmetrical distribution highlights the distinct regulatory responses induced by each protein. **C** KEGG pathway enrichment analysis of the identified DEGs, highlighting the top functional categories associated with M1 and NP expression

To investigate the differential impact of Influenza virus M1 and NP on the host transcriptome, we performed RNA-seq analysis in DF-1 cells expressing each protein. First, we identified differentially expressed genes (DEGs) by comparing each group to regular DF-1 cells (FDR < 0.05, |log2FC| ≥ 1). A total of 3,109 DEGs (724 shared + 2,385 unique) were identified in the NP-expressing group, whereas the M1-expressing group showed 1,594 DEGs (724 shared + 870 unique) (Fig. 1A). The overlap analysis revealed that only about 18% of the total DEGs were co-regulated, suggesting that NP expression induces a broader range of transcriptional alterations compared to that of M1, and both proteins largely modulate independent sets of host cellular pathways.

To further characterize the specific transcriptomic divergence, we performed a direct comparison between M1- and NP-expressing cells. The volcano plot revealed a remarkably symmetrical yet divergent distribution of DEGs (Fig. 1B). Under the threshold of nominal *p* < 0.05 and |log2FC| ≥ 1, a total of 7,304 genes showed significant differential expression between the two groups; 3,701 genes were significantly up-regulated in the M1 group (red), whereas 3,603 genes were up-regulated in the NP group (blue) (down-regulated in the M1 group). M1 induces stronger relative transcriptional shifts in a specific subset of genes when compared directly to NP.

Finally, KEGG pathway enrichment analysis was conducted to determine the functional implications of these distinct signatures (Fig. 1C). The genes preferentially expressed in the M1 group were predominantly associated with extracellular matrix (ECM)-receptor interaction and focal adhesion, suggesting that M1 primarily influences structural integrity and cellular morphology. In contrast, genes higher in the NP group were significantly enriched in cell cycle and DNA replication pathways. These results demonstrate that M1 and NP proteins utilize distinct molecular mechanisms to modulate the host cellular machinery: while M1 predominantly modulated pathways related to cellular structure, NP showed a higher impact on cell cycle and DNA replication pathways compared to M1, reinforcing the idea that NP plays a more critical role in interfering with host genetic stability (Additional files 1–5).

### Differential regulation of viral replication and host innate immune responses by M1 and NP expression

**Fig. 2:**
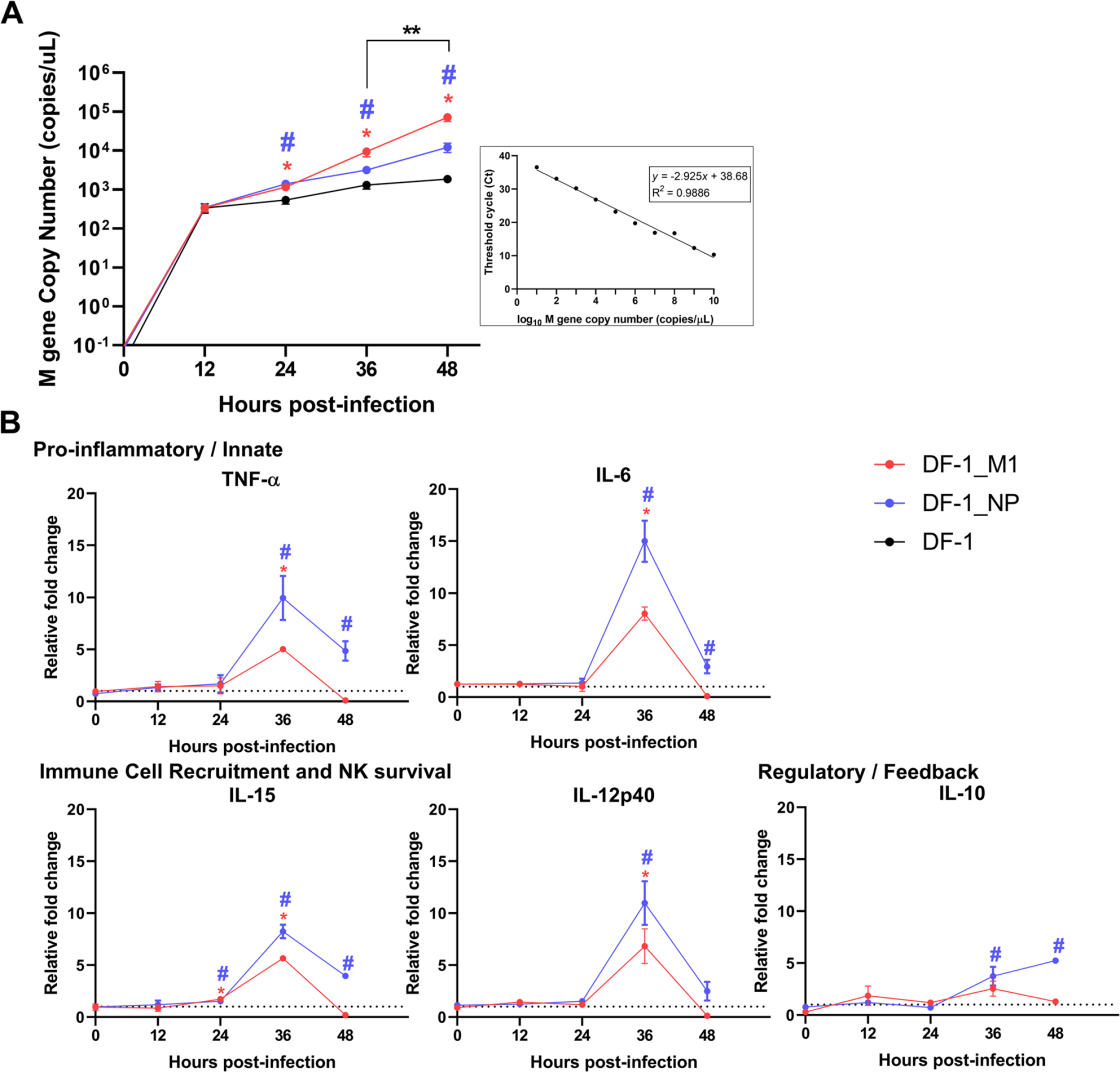
Differential regulation of viral replication and host immune responses by DF-1_M1 and DF-1_NP cells. **A** Absolute quantification of viral M gene copy numbers from 0 to 48 hours post-infection. The M gene copies were determined by RT-qPCR, and the inset displays the standard curve used for absolute quantification (*R*^*2*^ = 0.9886). **B** Relative fold changes in the mRNA expression levels of pro-inflammatory cytokines (TNF-α, IL-6), immune cell recruitment and NK survival factors (IL-15, IL-12p40), and a regulatory cytokine (IL-10). The dotted line (y = 1) represents the baseline expression level of the parental DF-1 control group. All data are presented as the mean ± standard deviation (SD) of three independent replicates. Significant increases (upregulation) compared to the parental DF-1 control group are indicated by * for DF-1_M1 and # for DF-1_NP (both p < 0.05). The bracket indicates a statistically significant difference directly between the DF-1_M1 and DF-1_NP groups (** *p* < 0.01)

To investigate the impact of stable M1 and NP protein expression on both viral growth kinetics and host-virus interactions, we first quantified the absolute copy number of the viral M gene over a 48-hour infection period. Up to 12 h post-infection (hpi), viral replication levels were comparable across all groups; however, starting from 24 hpi, both DF-1_M1 and DF-1_NP cells exhibited significantly accelerated viral replication compared to the parental DF-1 control (Fig. 2A). Notably, at the late stages of infection (36 and 48 hpi), DF-1_M1 cells demonstrated a significantly higher capacity for viral genome replication than DF-1_NP cells (** *p* < 0.01), reaching the highest M gene copy number at 48 hpi.

Alongside these viral kinetics, we evaluated the temporal mRNA expression profiles of key immune-related cytokines to understand the corresponding host innate immune responses. The induction of pro-inflammatory cytokines (TNF-α, IL-6), immune cell recruitment factors (IL-15, IL-12p40), and a regulatory cytokine (IL-10) peaked at 36 hpi in both transgenic cell lines before declining at 48 hpi (Fig. 2B). Interestingly, despite DF-1_M1 cells showing higher viral replication levels at these later stages, DF-1_NP cells triggered a higher cytokine response. At 36 hpi, the fold-changes of all tested cytokines in DF-1_NP cells were higher than those in DF-1_M1 cells.

Taken together, these results indicate that while both viral proteins facilitate viral replication compared to the control, they utilize distinct strategies: M1 provides a substantial advantage for sustained viral proliferation while maintaining a relatively moderated immune response, whereas the presence of NP renders the host cells highly sensitive to immune activation upon viral infection.

### NP accumulates in the culture supernatant of NP-expressing DF-1 cells (DF-1_NP)

**Fig. 3:**
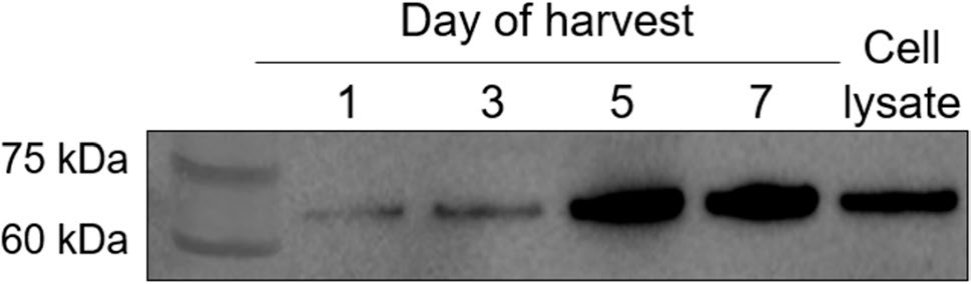
Detection of NP protein in supernatants of DF-1_NP. DF-1_NP cells were seeded and allowed to adhere for one day, after which the culture medium was replaced with Opti-MEM. Culture supernatants were collected at 1, 3, 5, and 7 days post-media exchange and analyzed by Western blotting. A whole-cell lysate from cells harvested at 1 day post medium exchange was included as a control. All samples were loaded at equal volumes (20 µL). Western blotting was performed using a polyclonal anti-Influenza A NP antibody (1:5000 dilution) and an HRP-conjugated mouse anti-rabbit IgG secondary antibody (1:5000 dilution). The specific bands were visualized using ECL

M1 and NP proteins are primarily localized within intracellular compartments of infected cells, although a small fraction of NP molecules has been reported on the surface of infected cells [20]. To assess extracellular protein secretion, culture media from DF-1_M1 and DF-1_NP cells were harvested at 1, 3, 5, and 7 days post-media exchange and analyzed by Western blotting. Under our experimental conditions, M1 protein was not detectable in the supernatant from DF-1_M1 cells. In contrast, NP protein was detectable in the culture supernatant from DF-1_NP cells at day 1 post-media exchange and increased notably at 5 and 7 post medium exchange (Fig. 3).

### Individual internal proteins (M1 and NP) of IAV induce independent humoral immune responses

**Fig. 4:**
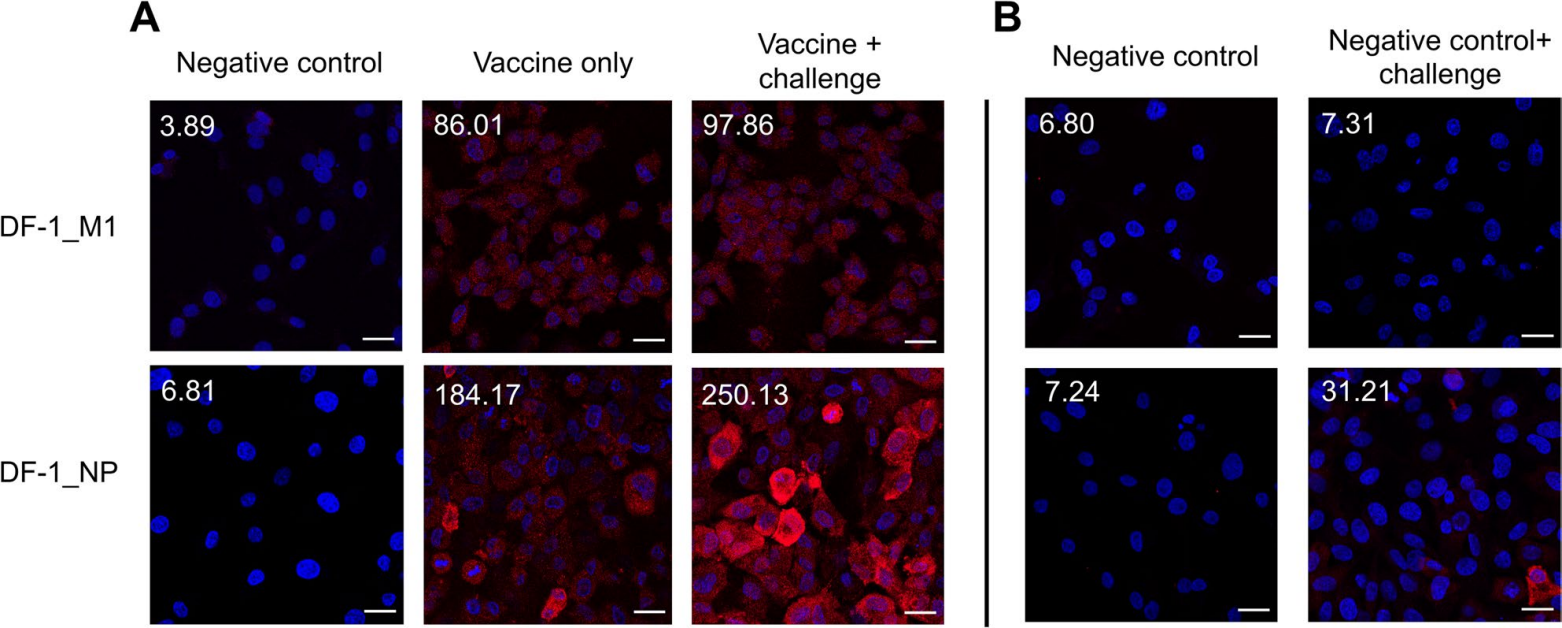
Humoral immune responses to M1 and NP induced by DF-1_M1 or DF-1_NP vaccination followed by SL20 challenge. **A** First independent experiment. DF-1 cells expressing either M1 or NP were incubated with chicken sera (1:500) derived from negative control, vaccination-only, or vaccination-plus-challenge groups. Bound antibodies were detected with Alexa Fluor™ 555-conjugated secondary antibody (1:1000), and nuclei were counterstained with DAPI. **B** Second independent experiment. The same procedure was repeated with sera from negative control and challenge-only groups to validate reproducibility of the observed patterns.Images were acquired using a Zeiss Axio Observer Z1/7 confocal microscope equipped with a 63×/1.40 oil immersion objective. Average fluorescence intensity per area is indicated in the top left corner of each image. The scale bar represents 10 μm

To determine whether viral infection following immunization with DF-1_M1 or DF-1_NP enhances humoral immunity to M1 or NP, serum samples were collected and analyzed by confocal imaging (Fig. 4). As primary antibodies, sera from non-immunized and immunized chickens were incubated with DF-1_M1 or DF-1_NP cells. In negative serum samples, fluorescence intensity was negligible, with values of 3.89 A.U. in DF-1_M1 cells and 6.81 A.U. in DF-1_NP cells (Fig. 4A, Negative control). The SL20-challenged, unvaccinated group showed slightly higher fluorescence intensities (7.31 A.U. and 31.21 A.U., respectively; Fig. 4B), but these remained lower than the vaccinated groups, supporting the advantage conferred by vaccination. In vaccinated groups, marked differences were observed. Chickens immunized with DF-1_M1 exhibited a baseline fluorescence intensity of 86.01 A.U., which modestly increased to 97.86 A.U. after challenge (Fig. 4A). In contrast, sera from DF-1_NP-immunized chickens showed a much higher baseline value of 184.17 A.U., which further increased substantially to 250.13 A.U. following challenge (Fig. 4A and Additional file 6).

### DF-1_M1 and DF-1_NP vaccines induce cellular immunity against the SL20 strain

**Fig. 5:**
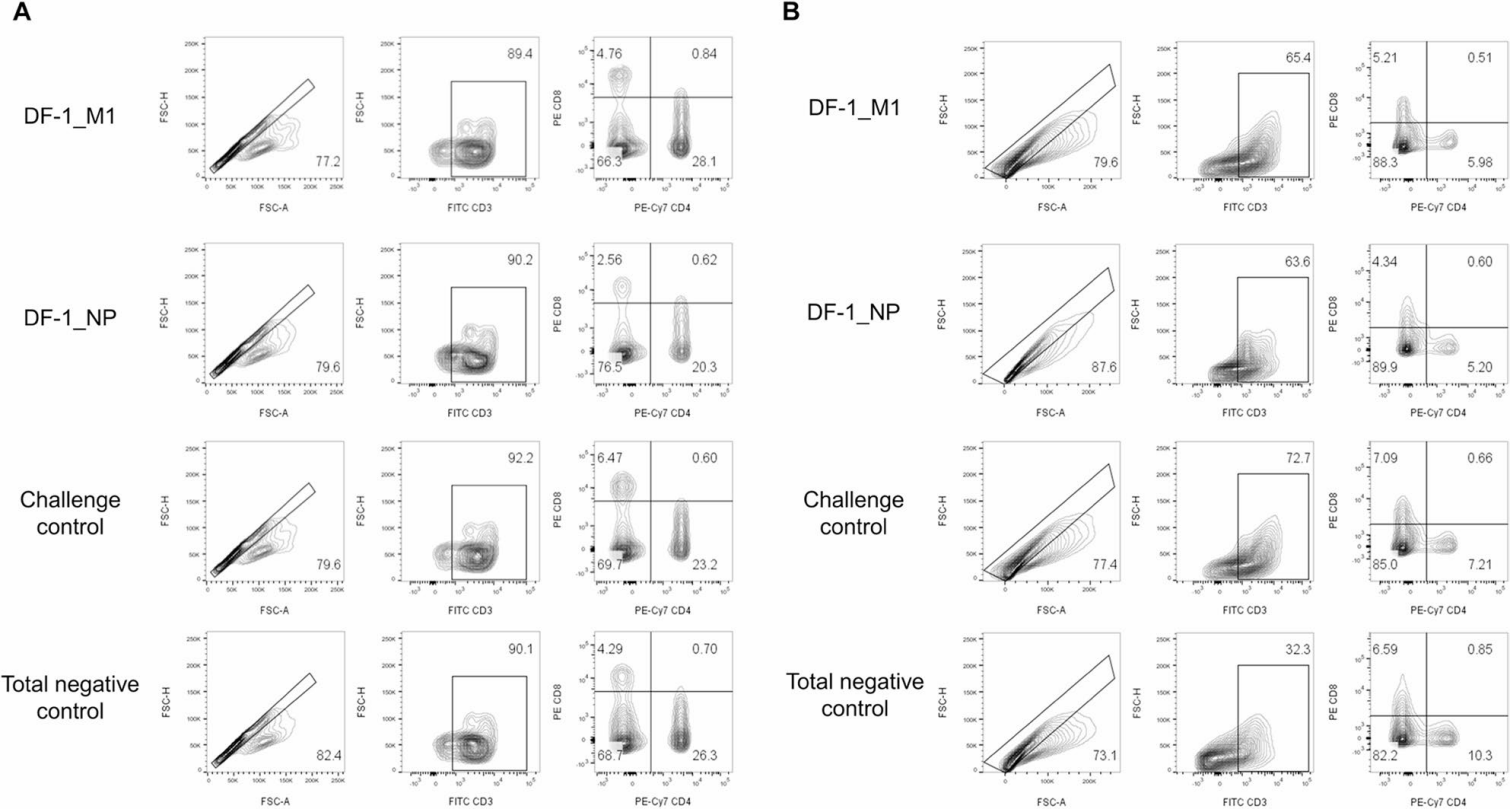
Flow cytometric analysis of CD8⁺/CD4⁺ T cell ratios in PBMCs and splenocytes following vaccination and viral challenge. Peripheral blood mononuclear cells (PBMCs) and splenocytes were collected from chickens vaccinated with DF-1_M1 or DF-1_NP, or left unvaccinated as challenge controls (N⁺) or negative controls (N⁻). Cells were gated sequentially on lymphocyte populations, CD3⁺ T cells, and then analyzed for CD4⁺ and CD8⁺ subsets. **A** Representative gating strategy and dot plots for PBMCs. **B** Representative gating strategy and dot plots for splenocytes

To evaluate the cellular immune responses induced by DF-1_M1 and DF-1_NP vaccines against the SL20 strain, flow cytometric analysis was performed using peripheral blood mononuclear cells (PBMCs) and splenocytes collected from vaccinated and control animals post viral challenge. CD8⁺/CD4⁺ T cell ratios were calculated for each group, along with the relative increase rates (%) compared to the negative control group (Table 1). Representative flow cytometry plots for gating strategy and T cell subset analysis are shown in Fig. 5. The challenge-control group (N⁺) exhibited the highest relative increase rates in both PBMCs (75.0%) and splenocytes (53.13%) compared with the vaccine groups. In PBMCs, DF-1_M1 induced only a modest increase (6.25%), whereas DF-1_NP resulted in a decrease relative to the negative control (–18.75%). In contrast, both vaccine groups showed clear increases in splenocytes (35.94% for DF-1_M1 and 29.69% for DF-1_NP). Overall, the vaccine groups elicited stronger responses in splenocytes than in PBMCs, whereas the challenge control showed the opposite trend.

**Table 1:** CD8⁺/CD4⁺ T Cell Ratios and Relative Increases Following DF-1 Cell–Based Vaccination and Challenge

Sample	Group	CD8^+^/CD4^+^	Relative increase rate (%)^a^
PBMC	DF-1_M1	0.17	6.25
	DF-1_NP	0.13	-18.75
	N+	0.28	75.00
	N-	0.16	0
Splenocyte	DF-1_M1	0.87	35.94
	DF-1_NP	0.83	29.69
	N+	0.98	53.13
	N-	0.64	0

Chickens were vaccinated with DF-1 cells stably matrix protein 1 (M1) or nucleoprotein (NP) of H9N2 influenza virus. N⁺ indicates unvaccinated but virus (SL20)-challenged controls; N⁻ indicates unvaccinated and unchallenged, total negative controls. CD8⁺/CD4⁺ T cell ratios were measured in PBMCs and splenocytes

^a^ Relative increase rate in comparison to N- group; DF-1_M1, M1-expressing DF-1 cell vaccine group; DF-1_NP, NP-expressing DF-1 cell vaccine group; N⁺, unvaccinated virus-challenge group; N⁻, unvaccinated unchallenged negative control group

### DF-1_M1 and DF-1_NP vaccines reduce viral shedding

To evaluate the protective efficacy of DF-1_M1 and DF-1_NP vaccines in reducing viral shedding, vaccinated and unvaccinated birds were challenged with the SL20 strain, and oropharyngeal and cloacal swabs were collected at 6- and 10- dpc. In the unvaccinated group, 4 out of 5 birds tested positive by oropharyngeal swab, whereas only 2 out of 5 birds in both the DF-1_M1 and DF-1_NP vaccinated groups tested positive (Table 1). The vaccine efficacy for both DF-1_M1 and DF-1_NP groups was 50%. All cloacal swab samples collected on 6-dpc were negative, along with oropharyngeal and cloacal swabs on 10-dpc.

**Table 2:** Protection efficacy of DF-1_M1 and DF-1_NP vaccines 6 days post-challenge

Vaccine	Number of positive (Ct) (*n* = 5)	Number of negative	Infection rate (%)*	Vaccine efficacy (%)**
DF-1_M1	2 (36.81, 37.19)	3	40	50
DF-1_NP	2 (35.86, 34.76)	3	40	50
Negative control	4 (33.69, 36.03, 34.12, 33.15)	1	80	-

*Infection rate: (Number of positive birds / Total birds tested) × 100%

**Vaccine efficacy: (1- (Infection rate in vaccinated group/infection rate in unvaccinated (negative) group) x 100%

## Discussion

This study evaluated the immunogenic properties of influenza virus internal proteins using a histocompatibility-matched chicken–cell vector system, demonstrating distinct transcriptional and immunological outcomes between M1 and NP. Our findings reveal that M1 primarily operates as a structural modulator to stabilize membrane organization, while NP serves as a regulator of host homeostasis, nuclear integrity, and innate immune evasion. These intrinsic differences fundamentally shape the downstream adaptive immune responses and protective efficacy.

M1 expression in DF-1 cells did not induce antiviral or stress-associated programs typically seen during influenza infection. Instead, the transcriptional changes were dominated by pathways related to membrane structure, solute transport, and cytoskeletal regulation. Moreover, M1 expression was associated with an increase in nutrient-transport and metabolic genes (e.g., SLC7A5, SLC34A1, and ME1). These changes may correspond to a shift toward glycolytic redirection and increased biosynthetic output, broadly consistent with the reported interaction of M1 with α-enolase and PKM2 to promote a Warburg-like metabolic pattern beneficial for viral assembly [21]. Pathways related to endosomal recycling and vesicle transport (e.g., ATP9A and LDLRAD3) were also affected [22], potentially reflecting an increased dependence on recycling endosomes for transporting viral materials. The STRIPAK–T108 phosphorylation axis provides a potential explanatory framework for these changes. Non-phosphorylated M1 interacts with PP2A-containing STRIPAK complexes to stabilize membrane-associated scaffolds, and our results suggest this pathway appears partially engaged even in the absence of other viral components [23–25]. The bias toward cytoplasmic and membranous compartments may be further influenced by the N242 substitution in the H9N2 M1 used in this study, which potentially weakens nuclear transport interactions compared to the SUMOylated K242 reported in other strains [26]. Collectively, RNA sequencing data suggests that M1 induces a “silent remodelling” phenotype, preparing the host for virion assembly by boosting metabolic throughput without triggering significant stress responses.

In contrast, NP reoriented host transcription toward a stress-adaptive and low-proliferative state by reducing key interferon-stimulated genes (e.g., IFIH1, MX1), consistent with its role in impairing innate detection through TRIM25 antagonism [27]. Beyond this direct suppression, NP maintains viral RNA integrity and limits the production of aberrant RNAs that serve as PAMPs (Pathogen-Associated Molecular Patterns). When NP is restricted—such as through targeted miRNA expression against NP mRNA—defective RNAs accumulate, activating RIG-I/MDA5 and inducing type I interferons [27]. This NP-dependent “viral genome protection” function is likely conserved among negative-sense RNA viruses and positions NP as a potential target for antiviral, immune-stimulating, or attenuation strategies.

A critical observation in our system was the spontaneous extracellular release of NP under non-replicative conditions, while M1 remained strictly intracellular. Earlier work using COS-1 cells reported that M1 alone could drive VLP formation [28]. However, our findings in the avian DF-1 system are consistent with subsequent studies showing that productive budding requires additional viral components like HA, NA, or M2 [29]. The extracellular release of NP is of particular interest as NP has been shown to engage TLR2 and TLR4, promoting inflammatory cytokine expression and NLRP3 inflammasome activation in human cell lines [30]. Although the strong fluorescence intensity observed in confocal microscopy for the DF-1_NP group partially reflects high antigen expression and antibody binding affinity rather than solely immunological function, this spontaneous release of NP in the absence of viral replication provides a plausible explanation for the stronger immune activation and enhanced humoral immunity observed in NP-expressing vaccine groups [20, 31].

While our vaccine platform primarily targets internal proteins that do not elicit traditional neutralizing antibodies (as measured by viral neutralization assays), the reduction in viral shedding confirms the presence of functional protective immunity. Furthermore, despite lower viral loads compared to M1, NP-expressing cells elicited a surge in pro-inflammatory and immune-recruiting cytokines (e.g., IL-15, IL-12p40). We hypothesize that this intense cytokine environment acted as a molecular adjuvant in vivo, potentiating antibody-mediated immunity in the DF-1_NP group while simultaneously inducing an antiviral state that restricted viral replication.

The CD8⁺/CD4⁺ T cell ratio is widely regarded as a biomarker of vaccine-induced cellular immunity [32]. In our study, both the DF-1_M1 and DF-1_NP vaccine groups displayed lower CD8⁺/CD4⁺ ratios after challenge compared to the challenge control group. Notably, the negative relative increase rate in the DF-1_NP group likely reflects the sequestration of activated effector T cells from the peripheral circulation to the respiratory tissues—the primary site of viral challenge [33].

This observation may be linked to antigen-specific regulatory mechanisms [34]. In murine influenza models, antigen-specific memory regulatory T cells (Tregs) accumulated more rapidly during secondary infection than primary infection, particularly in lung-draining lymph nodes and lung parenchyma. These Tregs effectively suppressed memory CD8⁺ T cell proliferation in an antigen-specific and MHC class II–dependent manner [35]. The functional protection observed, despite these modest peripheral ratios, suggests that vaccine-induced T cells were effectively recruited to target organs to exert their protective functions. Thus, antigen-specific memory Tregs may have been reactivated upon SL20 challenge, limiting CD8⁺ T cell expansion in the vaccine groups. Although we did not directly evaluate Tregs, prior reports indicate that chicken Tregs share comparable biological and immunoregulatory functions with their mammalian counterparts [35, 36].

The protective efficacy of individual M1 and NP antigens has been reported in several animal models, yet their relative contribution to immunity varies across species. In BALB/c mice, M1 DNA vaccination failed to confer protection, whereas NP DNA vaccination achieved complete protection via CD8⁺ T cell–mediated mechanisms [37, 38]. However, variations in murine MHC haplotypes and immune polarization profiles, such as the Th1-skewed response in C57BL/6 mice versus the Th2 bias in BALB/c mice, complicate direct application of these findings to other species [39]. In swine models, both M1- and NP-expressing viral vector vaccines were shown to reduce lung pathology following H1N1 challenge [40]. Interestingly, M1 conferred stronger protection despite the absence of detectable antibodies, indicating a role for non-humoral mechanisms. These findings emphasize the influence of host genetic background on antigen performance and underscore the value of our chicken host–cell vector model for dissecting antigen-specific immunity in avian species.

## Conclusion

In the present study, we highlight the differential immunogenicity of internal proteins, showing that NP elicits stronger immune activation than M1. This supports the concept that internal proteins can serve as promising vaccine antigens. Consistently, in our earlier work, PBMCs from chickens immunized with DF-1 cells transiently expressing M1 or NP exhibited increased interferon-γ production upon viral restimulation [19], indicating that stable expression is not a prerequisite for eliciting cellular responses.

Thus, while DF-1_M1 or DF-1_NP alone may not provide total sterile immunity, the DF-1–based host–vector system provides a unique advantage by enabling antigenic evaluation of individual viral genes without interference from whole-virus components. Overall, this work reveals how internal viral proteins employ divergent cellular strategies to influence immune outcomes, bridging molecular mechanisms with vaccine design. Moving forward, exploring the synergistic effects of combining M1 and NP, along with other conserved internal antigens, will be a crucial next step toward developing a more potent and broadly protective universal influenza vaccine for poultry.

## Methods

### Ethical statement and animal husbandry

All animal experiments were conducted in accordance with the ARRIVE guidelines 2.0 and institutional guidelines, approved by the Animal Care Committees of Seoul National University (SNU-250508-3-1, approved on July 10th, 2025). Female specific-pathogen-free (SPF) VALO chickens were purchased from VALO Biomedia GmbH (Osterholz-Scharmbeck, Germany). The birds had a mean body weight of approximately 210 ± 20 g at the start of the experiment (3 weeks of age). All animals were housed in negative-pressure isolators under appropriate husbandry conditions (temperature: 20–25 °C; humidity: 40–60%; light/dark cycle: 12/12 h) with feed and water provided *ad libitum*. Animals were monitored twice daily for clinical signs of illness or distress. Humane endpoints were defined as severe weight loss (> 20%), inability to eat or drink, or severe lethargy, at which point animals would be humanely euthanized by CO2 inhalation.

### Cells, plasmids, and chemical reagents

The M1 and NP genes of the A/chicken/South Korea/SL20/2020 (H9N2, Y280-lineage) strain were cloned into the lentiviral vector pLVX-puro. To generate stable DF-1 cell lines (ATCC CRL-3586™, Manassas, VA, USA), we utilized the 4th-generation Lenti-X™ Packaging Single Shots (VSV-G) system (Takara Bio Inc., Shiga, Japan). This system is designed to be replication-incompetent, ensuring that once the host DF-1 cells are transduced, no progeny viruses are produced. Transduced cells were selected using puromycin to establish permanent lines, designated DF-1_M1 and DF-1_NP. To ensure experimental consistency and a controlled transfection dose, the lentivirus was used directly from the initial production without any further passage.

### Transcriptomic profiling

Total RNA was extracted from confluent DF-1_M1 and DF-1_NP cells cultured in T175 flasks using TRIzol reagent (Invitrogen, Carlsbad, CA, USA). Raw sequencing reads were subjected to adapter and quality trimming using Trimmomatic v0.38, and aligned to the *Gallus gallus* reference genome (bGalGal1.mat.broiler.GRCg7b) using HISAT2 v2.1.0. Transcript abundances were quantified in reference-guided mode with StringTie v2.1.3b to generate gene expression profiles, including Read Counts, FPKM, and TPM. Normalization and differential expression analysis were performed with edgeR v3.40.2, employing the exactTest function. Multiple testing correction was conducted using the Benjamini–Hochberg procedure to control the false discovery rate (FDR). Genes with FDR < 0.05 and |log2 Fold Change| ≥ 1 (corresponding to |fold change| ≥ 2) were considered significantly differentially expressed genes (DEGs). To further characterize the divergence between M1 and NP systems, a direct differential analysis (DF-1_M1 vs. DF-1_NP) was performed using the interaction model within edgeR to identify genes uniquely modulated by each viral protein.

### Functional enrichment analysis and visualization

Functional enrichment was performed to identify the biological significance of the DEGs. Gene Ontology (GO) enrichment analysis was conducted using g: Profiler (v e113_eg59_p19_f6a03c19). For KEGG pathway enrichment, we employed the Fisher’s Exact Test and hypergeometric distribution via the clusterProfiler package (v4.6.0) in R. Statistically significant pathways were identified based on an adjusted *P*-value < 0.05 and visualized using Dot Plots (Fig. 5C), where the dot size represents the gene ratio and the color indicates the significance level (adjusted *P*-value). Due to the limited availability of comprehensively ranked gene set references specifically for the avian (*Gallus gallus*) model, this Over-Representation Analysis (ORA) was selected over Gene Set Enrichment Analysis (GSEA) to ensure biological accuracy.

To visualize the transcriptomic divergence and functional distributions, volcano plots and Venn diagrams were generated using RStudio (v2023.12.1) with the ggplot2 package. Specifically, the volcano plot was constructed using nominal *P*- values to illustrate the global distribution of gene expression changes, while the Venn diagram was based on stringent DEGs filtered by FDR < 0.05. All visualizations and statistical interpretations were integrated to characterize the distinct host responses induced by M1 and NP expression.

### Viral replication and cytokine expression analysis

To assess the functional impact of M1 or NP pre-expression on viral susceptibility, DF-1_M1, DF-1_NP, and parental DF-1 cells were infected with H9N2 (SL20) virus at a multiplicity of infection (MOI) of 0.2. The infection medium was supplemented with a minimal concentration of TPCK-treated trypsin (0.1 µg/mL) to facilitate viral cleavage. Owing to the high sensitivity of DF-1 fibroblasts to TPCK-treated trypsin and virus-induced cytopathic effects (CPE), significant cell detachment and lysis occurred within 48 h post-infection. Consequently, supernatants containing detached cells and cellular debris were collected at 12-hour intervals up to 48 h post-infection, and stored at -80 °C to capture both extracellular viral progeny and associated host transcripts. Viral loads were quantified by measuring M gene copy numbers using RT-qPCR (Median Diagnostics Inc., Chuncheon, Republic of Korea).

A standard curve was generated using a plasmid containing the H9N2 M gene, with copy numbers calculated according to Avogadro’s number and plasmid molecular weight. Additionally, host cytokine expression profiles (GAPDH, TNF-α, IL-6, IL-15, IL-12p40, IL-10, IFN-γ, IL-2, IL-4, perforin and granzyme) were analyzed from the same supernatants using the HS One-step RT-qPCR 2X Master Mix (Elpisbiotech, Republic of Korea). All assays were performed in triplicate, and relative fold changes were calculated using the 2^−ΔΔCt^ method [41–45] (Additional file 7).

### Western blotting and Enhanced Chemiluminescence (ECL)

To assess the presence of M1 or NP proteins in the culture supernatant, DF-1_M1 and DF-1_NP cells were seeded into 6-well plates at a density of 0.3 × 10⁶ cells per well. After 24 h, the medium was replaced with Opti-MEM (Gibco; Thermo Fisher Scientific, Waltham, MA, USA) to replicate the conditions used for vaccination. Culture supernatants were collected on days 1, 3, 5, and 7 post-seeding. For comparison, a whole-cell lysate was prepared 24 h after seeding using radioimmunoprecipitation assay (RIPA) buffer (Invitrogen, Waltham, MA, USA) and analyzed alongside the supernatants.

Enhanced chemiluminescence (ECL) detection was performed prior to standard Western blot analysis on the same membrane. Briefly, membranes were incubated with either serum collected from influenza virus vaccine–immunized animals in our previous study (used as the primary antibody for M1 detection) or polyclonal anti-influenza A nucleoprotein (NP) primary antibody (Invitrogen, Waltham, MA, USA) at a 1:5000 dilution, followed by a horseradish peroxidase (HRP)-conjugated mouse anti-rabbit IgG secondary antibody (Santa Cruz Biotechnology, Dallas, TX, USA) at a 1:5000 dilution. Membranes were then incubated with WesternBright™ Quantum substrate (Advansta, San Jose, CA, USA), and chemiluminescent signals were visualized using an ImageQuant™ LAS 4000 imaging system (GE Healthcare Life Sciences, Chicago, IL, USA). Following ECL imaging, membranes were washed with Tris-buffered saline containing 0.1% Tween-20 for approximately 2 h at room temperature, and HRP-based chromogenic development was subsequently performed. Molecular weight markers from the chromogenic (HRP) images were overlaid onto the ECL images for clarity.

### B21 haplotype determination

To match the MHC haplotype of the DF-1 cell line (B21), VALO SPF chickens were genotyped by LEI0258 PCR as described previously [46]. Chickens exhibiting banding patterns identical to the B21 haplotype upon gel electrophoresis were selected for experimentation. In a preliminary study, we confirmed that this approach provides reliable genotyping accuracy within a controlled chicken population [19]. Because DF-1 cells carry the B21 haplotype, this strategy ensured that the chickens recognized DF-1 cells as “self,” thereby allowing immune responses to be directed specifically toward the expressed viral antigens rather than against allogeneic cell components.

From the screened population, a total of 20 chickens were enrolled. Sample size was determined based on previous studies of similar design, as no formal power calculation was performed. The selected chickens were randomly assigned to four groups (*n* = 5 per group; DF-1_M1 vaccinated group, DF-1_NP vaccinated group, challenge control, total negative control).

### Cell vector vaccine preparation, vaccination and challenge experiment

Although longer culture periods increased protein concentrations in the supernatant, cellular viability was prioritized for vaccine preparation. Three-week-old VALO SPF chickens were intramuscularly vaccinated with 2 × 10^6^ live DF-1_M1 or DF-1_NP cells. The vaccination dose was determined based on our preliminary studies using transient transfection, which confirmed that this cell number is sufficient to elicit an immunogenic response [19]. Cell concentration was standardized using live cell counts (Trypan blue exclusion method) prior to administration. The vaccine formulation consisted of the cell suspension in Opti-MEM without the addition of any adjuvant. Chickens were boosted two weeks later, and challenged with the SL20 virus one-week post-boost.

The A/chicken/South Korea/SL20/2020 (H9N2, Y280-lineage) strain was isolated from oropharyngeal swabs of native chickens at a live bird market in South Korea [47]. The virus was propagated in specific-pathogen-free (SPF) embryonated chicken eggs (ECEs) (VALO Biomedia GmbH, Osterholz-Scharmbeck, Germany) and used for intranasal challenge at a dose of 10^6^ EID₅₀/0.1 mL.

Oropharyngeal and cloacal swabs were collected at 6- and 10-days post-challenge (dpc) and tested by RT-qPCR (Median Diagnostics Inc., Chuncheon, Republic of Korea).

### Confocal microscopy

DF-1_M1 and DF-1_NP cells cultured on coverslips were fixed with 4% paraformaldehyde (Sigma-Aldrich, MO, USA) and permeabilized using 0.2% Triton X-100 in PBS supplemented with 2% normal goat serum (Jackson ImmunoResearch, PA, USA). Cells were incubated with a primary antibody (1:500 dilution) using sera from experimental chickens. Sera were collected at 10-dpc (or the equivalent time point for total negative controls) from birds that received a prime-boost vaccination regimen followed by viral challenge. Subsequently, cells were incubated with an Alexa Fluor™ 555-conjugated secondary antibody (1:1000 dilution; Thermo Fisher Scientific, MA, USA). Nuclei were counterstained with DAPI (BioLegend, CA, USA), and fluorescence images were acquired using an Axio Observer Z1/7 microscope with a 63×/1.40 oil immersion objective (Zeiss, Germany) operated via ZEN 3.8 software.

### Flow Cytometry (FACS) analysis

Flow cytometry was performed using the SH800S system (Sony Biotechnology, San Jose, CA, USA) to evaluate immune cell populations in peripheral blood mononuclear cells (PBMCs) and splenocytes. PBMCs and splenocytes were isolated from whole blood using Lymphoprep™ density gradient medium (STEMCELL Technologies, Vancouver, Canada) 10-dpc. Following isolation, cells were stained with anti-chicken CD3-FITC, CD4-APC, and CD8α-PE antibodies (SouthernBiotech, Birmingham, AL, USA), and data were acquired under consistent instrument settings. T cells were gated on CD3⁺ expression, after which CD4⁺ and CD8α⁺ subsets were analyzed to determine relative population dynamics.

### Statistical analysis

Statistical analysis for transcriptomic data was performed using edgeR (v3.40.2) and R software. For virus shedding and FACS analysis, individual data points are presented directly in tables to show biological variability. For signal intensity analysis, descriptive statistics (mean ± standard deviation) were calculated using Microsoft Excel. For the RT-qPCR data, including the absolute quantification of viral M gene copy numbers and the relative fold changes in cytokine mRNA expression, statistical significance between groups at each time point was determined using an unpaired two-tailed Student’s *t*-test. (IBM Corp. Released 2021. IBM SPSS Statistics for Windows, Version 28.0. Armonk, NY: IBM Corp.). *P*-values less than 0.05 were considered statistically significant.

## Supplementary Information

Additional file 1. Full transcriptomic profile and differential expression analysis of M1- and NP-expressing DF-1.

Additional file 2. Fold change values and raw P-values of significant DEGs used for downstream functional enrichment.

Additional file 3. Summary of statistically significant KEGG biological pathways enriched in M1- and NP-expressing groups.

Additional file 4. Detailed gene lists associated with each significantly enriched KEGG pathway.

Additional file 5. Gene Ontology (GO) Enrichment Analysis of Biological Process, Molecular Function, and Cellular Component.

Additional file 6. Quantitative analysis of vaccine-induced immune responses. Raw data containing area and intensity values representing antibody responses induced by M1 and NP vaccines, including measurements post-viral challenge.

Additional file 7. Primers used in this study. List of oligonucleotide primers and sequences used for the cloning and construction of M1 and NP expression vectors, along with RT-qPCR primers.

## Data Availability

Data is provided within the manuscript or in Additional files.

## Abbreviations

IAV: Influenza A virus
NP: Nucleoprotein
M1: Matrix protein 1
CTL: Cytotoxic T lymphocytes
hpi: Hours post-infection
dpc: Days post-challenge
